# Recent advances in understanding *Pseudomonas aeruginosa *as a pathogen

**DOI:** 10.12688/f1000research.10506.1

**Published:** 2017-07-28

**Authors:** Jens Klockgether, Burkhard Tümmler

**Affiliations:** 1 Molecular Pathology of Cystic Fibrosis Clinical Research Group, Clinic for Paediatric Pneumology, Allergology, and Neonatology, OE 6710, Hannover Medical School, Hannover, Germany; 2 Biomedical Research in Endstage and Obstructive Lung Disease Hannover (BREATH), Member of the German Centre for Lung Research, Hannover, Germany

**Keywords:** Pseudomonas aeruginosa, type III secretion system, T3SS, type VI secretion system, T6SS, c-di-GMP

## Abstract

The versatile and ubiquitous *Pseudomonas aeruginosa *is an opportunistic pathogen causing acute and chronic infections in predisposed human subjects. Here we review recent progress in understanding *P. aeruginosa* population biology and virulence, its cyclic di-GMP-mediated switches of lifestyle, and its interaction with the mammalian host as well as the role of the type III and type VI secretion systems in *P. aeruginosa* infection.

## Introduction

*Pseudomonas aeruginosa* is a metabolically versatile ubiquitous gamma-proteobacterium that thrives in soil and aquatic habitats and colonizes the animate surfaces of plants, animals, and humans ^1^. *P. aeruginosa* may cause multiple infections in man that vary from local to systemic and from benign to life threatening. During the last few decades, the cosmopolitan Gram-negative bacterium has become one of the most frequent causative agents of nosocomial infections associated with substantial morbidity and mortality ^2^. Pneumonia and sepsis in intensive care unit (ICU) patients still have a bleak prognosis ^3^. Chronic airway infections with *P. aeruginosa* are a major cause of morbidity in people with cystic fibrosis (CF) or chronic obstructive pulmonary disease (COPD) ^3– 5^. Here we report on recent advances in the understanding of host–pathogen interactions with particular emphasis on infections with *P. aeruginosa* in humans.

## Differential pathogenicity of clone types

**Figure 1.**
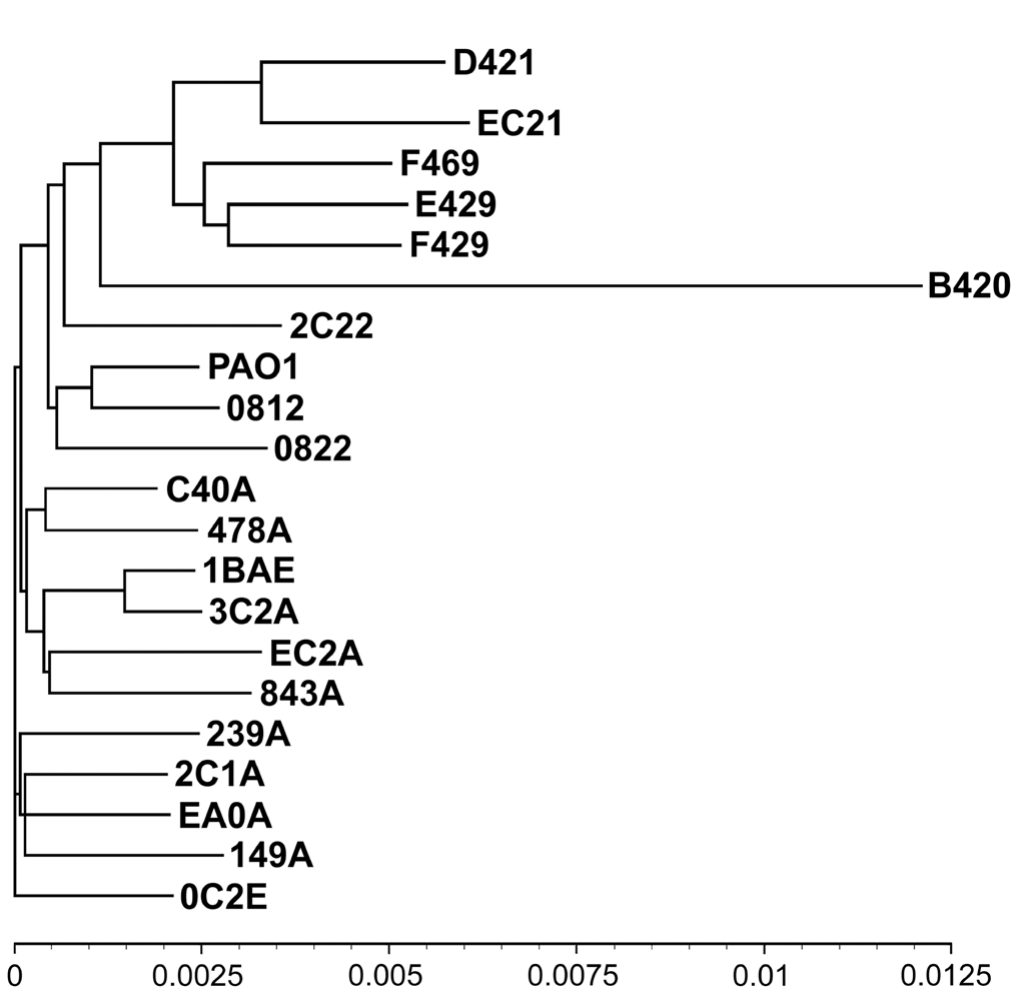
Phylogenetic tree of *Pseudomonas aeruginosa* strains representing the 20 most common clones in the global *P. aeruginosa* population ^8, 9^. Clones are designated by a hexadecimal code representing single nucleotide polymorphisms (SNPs) at seven conserved loci of the core genome and the subtypes of *exoU*/ *exoS* and *fliC* ^9^. The B420 clone represents the PA7-outlier group, D421, EC21, F469, E429, and F429 the ExoU-positive clade, and all other clones the ExoS-positive clade. The tree is based on paired genome-wide comparisons of SNPs in the core genome ^8^. The scale indicates the sequence diversity. Reproduced from Figure 5 of 8.

*P. aeruginosa* is equipped with a large repertoire of virulence determinants ( Table 1) and a complex regulatory network of intracellular and intercellular signals ^6, 7^ that allow the bacteria to adapt to and thrive in an animate niche and to escape host defence. This ability, however, varies from clone to clone and from strain to strain in the worldwide *P. aeruginosa* population. When representative isolates of the 20 most common clones in clinical and environmental habitats ( Figure 1) were tested in three established infection models, i.e. an acute infection of murine airways, lettuce, and wax moth larvae, an unexpected gradient of pathogenicity was observed ^8^. Under the conditions of the standardized acute airway infection model in the mouse, the full spectrum of possible host responses to *P. aeruginosa* was seen that ranged from unimpaired health to 100% lethality. Likewise, similar gradients of virulence were observed with plant and insect hosts whereby the pathogenicity of most strains differed by host. A strain could be innocuous for the mouse but highly pathogenic for the lettuce, and vice versa.

**Table 1.** Virulence effectors of *Pseudomonas aeruginosa* ^*^.

Name	Activity and function
ArpA	Alkaline protease, zinc metalloprotease; degrades host immune complements C1q, C2, and C3 and cytokines interferon (IFN)-γ and tumour necrosis factor (TNF)-α
Cif	Cystic fibrosis transmembrane conductance regulator (CFTR) inhibitory factor, epoxide hydrolase; promotes sustained inflammation by hydrolysing the paracrine signal 14,15-epoxyeicosatrienoic acid that stimulates neutrophils to produce the proresolving lipid mediator 15-epi lipoxin A _4_; Cif increases the ubiquitination and lysosomal degradation of some ATP-binding cassette transporters (ABC) including CFTR, P-glycoprotein, and TAP1
ExlA	Exolysin A, a pore-forming toxin that induces plasma membrane rupture in epithelial, endothelial, and immune cells
ExoS	Bifunctional toxin with Rho GTPase-activating protein (RhoGAP) activity and ADP-ribosyltransferase (ADPRT) activity; it blocks the reactive oxygen species burst in neutrophils by ADP-ribosylation of Ras, thereby preventing the activation of phosphoinositide-3-kinase (PI3K), which is required to stimulate the phagocytic NADPH-oxidase
ExoT	Bifunctional toxin with RhoGAP activity and ADPRT activity; it impairs the production of reactive oxygen species burst in neutrophils and promotes the apoptosis of host cells by transforming host protein Crk by ADP-ribosylation into a cytotoxin and by activation of the intrinsic mitochondrial apoptotic pathway
ExoU	Phospholipase A _2_, releases fatty acids from a broad range of phospholipids and lysophospholipids; it becomes activated by interaction with ubiquitin or ubiquitinylated proteins in the cytosol of the host cell
ExoY	Nucleotidyl cyclase with preference for cGMP and cUMP production; it becomes activated by binding to filamentous actin
LasA	Zinc metalloprotease of the M23A family; it enhances elastolytic activity of LasB
LasB	Zinc metalloprotease with strong elastolytic activity
PlcH	Haemolytic phospholipase C that releases phosphate esters from sphingomyelin and phosphatidylcholine
PlcN	Non-haemolytic phospholipase C that releases phosphate esters from phosphatidylserine and phosphatidylcholine
PldA	Trans-kingdom toxin, phospholipase D, facilitates intracellular invasion of host eukaryotic cells by activation of the PI3K/ Akt pathway
PldB	Trans-kingdom toxin, phospholipase D, facilitates intracellular invasion of host eukaryotic cells by activation of the PI3K/ Akt pathway
PrpL	Class IV protease, lysine endoproteinase, degrades proteins such as complement, immunoglobulins, elastin, lactoferrin, and transferrin
Pyocyanin	Redox-active zwitterion that is cytotoxic
Rhamnolipids	Chemically heterogeneous group of monorhamnolipids and dirhamnolipids that are also biosurfactants and cause haemolysis and lysis of immune effector cells
Tox A	Exotoxin A, a toxin with ADPRT activity; it mediates its entry into target host cells through its cell-binding domain, then ADP-ribosylates host elongation factor 2 (EF-2) to block protein synthesis through its enzymatic domain
TplE	Trans-kingdom toxin, phospholipase A1, disrupts the endoplasmic reticulum and thereby promotes autophagy by the activation of the unfolded protein response

^*^The Table lists proven virulence effectors in infections of mammals

The differential genetic repertoire of clones maintains a host-specific gradient of virulence in the *P. aeruginosa* population whereby differential sets of pathogenicity factors and mechanisms are employed to conquer the diverse animate niches. In other words, the fitness of a clone to colonize and to persist differs by habitat. This conclusion is supported by real-world data. Close to 3,000 spatiotemporally unrelated isolates from the environment, acute human infection, and chronically colonized COPD and CF airways were genotyped with a marker microarray yielding 300-odd clone genotypes ^9^. The 20 most frequent clones had an absolute share of 44%, indicating that the *P. aeruginosa* population is dominated by few epidemic clonal lineages. The most abundant clones like C or PA14 were detected in all habitats, albeit at different frequencies ^10^. On the other hand, the proportion of habitat-specific clones was 25% in COPD, 32% in acute infections, 39% in the environment, and 54% in CF, indicating that the CF lungs select for rare clones that can withstand exposure to a hostile immune system and regular antimicrobial chemotherapy and that the soil and aquatic habitats accommodate a subgroup of clones that cannot colonize a human niche. The spectrum of clones was broader in COPD and CF lungs than in the multiple niches of acute infections, implying that the airways of a predisposed host can be colonized by more clone types than the organs of a previously healthy and immunocompetent host that suffers from an acute insult. In summary, a human habitat can be colonized by some generalists like clone C or PA14 and some minor clones that have a low prevalence in the global population but are endowed with clone-specific features of fitness and/or pathogenicity to become a dominant member in the particular human niche. Since most research has focused on clinical isolates, the analysis of isolates from non-clinical habitats may reveal features yet unknown for *P. aeruginosa.*

## Non-coding RNAs

The Pseudomonas genome database ^11^ (20 June 2017) lists 37 non-coding RNAs in the genome of reference strain PAO1. These small RNAs (sRNA) have regulatory functions. Prominent examples are RsmY, RsmZ, or CrcZ, which are master regulators of bacterial lifestyle, biofilm formation, and carbon metabolism ^12– 15^. Recently, the first example of trans-kingdom biological activity of a regulatory *P. aeruginosa* sRNA was described ^16^. *P. aeruginosa* bacteria released outer membrane vesicles (OMVs) containing thousands of unique sRNA fragments. The OMVs fused with and delivered sRNAs into mammalian cells, thereby attenuating neutrophil recruitment and the secretion of pro-inflammatory cytokines.

## Type III and type VI secretion systems

The type III secretion system (T3SS) and its effectors are the major virulence determinants of *P. aeruginosa* ^17^. The T3SS forms a needle that directly injects virulence effectors (ExoS, T, U, and Y) into the host cell. The ADP-ribosyltransferase (ADPRT) ExoS ^18, 19^ and the phospholipase A2 ExoU ^20, 21^ occur almost mutually exclusively in *P. aeruginosa* so that the population is currently differentiated into a major ExoS-positive clade, a minor ExoU-positive clade, and a minute T3SS-negative clade lacking both ExoS and ExoU ^9, 22^. ExoU and its homologue PlpD ^23, 24^, which is secreted through the type V secretion system, are lipolytic enzymes of the patatin-like protein family. ExoU is highly cytotoxic and more virulent than ExoS in infection models, which correlates with the higher morbidity of acute infections with ExoU-positive than with ExoS-positive strains. ExoS and ExoT consist of an N-terminal GTPase-activating protein (GAP) domain and a C-terminal ADPRT domain. Both ExoS and ExoT disrupt the signalling pathway responsible for the activation and assembly of the phagocytic NADPH oxidase and thereby block the production of reactive oxygen species ( Table 1) ^25^. ExoT moreover promotes the apoptosis of host cells. The GAP domain of ExoT triggers the mitochondrial intrinsic pathway of apoptosis ^26^, and ADPRT domain activity transforms the focal adhesion adaptor protein Crk of the host cell into a cytotoxin that induces a form of programmed cell death known as anoikis, which occurs in cells when they detach from the surrounding extracellular matrix ^27^. The pathogenicity of ExoS, ExoU, and ExoT is well established, but the role of ExoY during infections still needs to be elucidated ^28– 31^. ExoY synthesizes numerous cyclic nucleotides (cNMPs) ^32^. The uncommon cUMP turned out to be the most prominent cNMP generated in the lungs of mice infected with ExoY overexpressing *P. aeruginosa.* cUMP was detectable in body fluids, suggesting that this unusual cNMP in contrast to cAMP or cGMP is not rapidly degraded and thus may interfere with the second messenger signalling of the host.

The T3SS-negative clade represents three groups of taxonomic outliers with above-average sequence diversity ^8, 22, 33^. This, however, does not imply that these strains are innocuous. Firstly, isolates have transferred genomic islands to major ExoS-positive clonal lineages and thereby supplied the recipients with determinants of resistance to antimicrobials ^34^. Secondly, some strains harbour the recently discovered two-partner secretion system ExlAB ^35, 36^. ExlB exports a pore-forming toxin called exolysin (ExlA), which induces plasma membrane rupture in epithelial, endothelial, and immune cells but not in erythrocytes. Thirdly, these outliers, like all other ExoS- or ExoU-positive *P. aeruginosa* strains, possess type VI secretion systems (T6SS).

The T6SS is a bacterial nanomachine that shares similarity with the puncturing device of bacteriophages ^37, 38^. The three separate H1-, H2-, and H3-T6SS translocate proteins between cells by a mechanism analogous to phage tail contraction. The H1-T6SS delivers the three toxins Tse1–3, which kill bacterial competitors inhabiting the same niche. The H2- and H3-T6SS target both prokaryotic and eukaryotic cells, i.e. they translocate trans-kingdom effectors ^39– 44^. These toxins exert antibacterial activity and facilitate intracellular invasion of host eukaryotic cells ( Table 1). The phospholipase TplE, for example, disrupts the endoplasmic reticulum and thereby promotes autophagy by the activation of the unfolded protein response ^40, 42– 44^.

## Control of lifestyle by cyclic di-GMP signalling

*P. aeruginosa* can switch between a motile and a sessile lifestyle and can modulate the secretion of virulence effectors by a plethora of transcription factors, two-component systems, non-coding RNAs, and quorum-sensing networks ^6, 45^. Research in the last few years has focused on the complex signalling pathways mediated by the second messenger cyclic di-GMP (c-di-GMP) ^46, 47^. *P. aeruginosa* possesses more than 40 diguanylate cyclases and c-di-GMP-degrading phosphodiesterases, the specificities of which are currently under investigation ^46, 47^.

Elevated levels of c-di-GMP promote biofilm formation typical for chronic infections and repress flagellum-driven swarming motility typical for acute infections and vice versa. The master transcription regulator FleQ, for example, activates the transcription of flagellar genes when intracellular c-di-GMP levels are low, but upon binding of c-di-GMP FleQ converts to an activator of the expression of genes involved in biofilm formation such as the adhesin CdrAB or the exopolysaccharides Psl and Pel ^48, 49^. The binding of c-di-GMP leads to major conformational rearrangements in FleQ, which reasonably explains the dual role of FleQ in promoting both the sessile and the motile lifestyles dependent on c-di-GMP levels.

The third major exopolysaccharide in *P. aeruginosa* biofilms is alginate, a polymer of mannuronic and guluronic acid. Alginate secretion is promoted by the protein Alg44 upon the binding of two c-di-GMP molecules ^50^. The production of alginate is induced under microaerophilic or anaerobic conditions, as is typically the case for chronic infections of COPD and CF lungs ^51^. A recently discovered three-gene operon, *sadC-odaA-odaI*, controls the oxygen-dependent synthesis of alginate ^52^. During anaerobiosis, the diguanylate cyclase SadC produces c-di-GMP, but in the presence of oxygen OdaI inhibits c-di-GMP synthesis by SadC ^52^. Besides the induction of alginate biosynthesis, SadC inhibits swimming, swarming, and twitching motility, promotes the production of the exopolysaccharide Psl, and is a member of the RetS/LadS/Gac/Rsm regulatory network that constitutes the decision-making switch between sessile and motile lifestyles ^53, 54^.

The transition from the biofilm to the planktonic growth state is under the complex regulatory control of many other players influencing the intracellular c-di-GMP levels. The chemosensory protein BdlA, the diguanylate cyclase GcbA, and the c-di-GMP phosphodiesterases DipA, RbdA, and NbdA are known proteins that are required for dispersion to occur ^55– 57^.

## Within-host evolution of *P. aeruginosa*

*P. aeruginosa* may persist for decades in the lungs of individuals with CF, which provides the rare opportunity to study the within-host evolution of a bacterial pathogen for an extended period of time ^58^. A single case who acquired *P. aeruginosa* in the neonatal period has already been analysed by whole genome sequencing of serial *P. aeruginosa* isolates more than 10 years ago ^59^. Subsequently, the genetic adaptation of *P. aeruginosa* to CF lungs has been investigated in carriers of major clones ^60^ or transmissible lineages ^61– 65^ and, more recently, in a cohort of CF children who were followed for the early phase of infection ^66^. This study of 474 longitudinal isolates from 34 CF patients seen at the Copenhagen CF clinic, representing 36 different clonal lineages of *P. aeruginosa*, showed evidence for positive selection at 52 genes, suggesting adaptive evolution to optimise pathogen fitness. Numerous genes encode traits known to be involved in CF lung infection, such as antibiotic resistance or biofilm formation. In our own ongoing work on serial *P. aeruginosa* isolates from patients seen at the Hannover CF clinic, we rediscovered just a quarter of these 52 candidate pathoadaptive genes to be frequently mutated, demonstrating the versatility of *P. aeruginosa* to conquer and persist in CF airways. This versatility also shows up in an extensive phenotypic diversity within the *P. aeruginosa* populations inhabiting CF airways. The analysis of 15 variable traits in 1,720 isolates collected from 10 carriers of the Liverpool epidemic strain revealed 398 unique subtypes ^67^. In summary, all of these cross-sectional and longitudinal studies demonstrated extensive genetic and phenotypic diversity of the *P. aeruginosa* populations in CF lungs ^58– 72^.

## The response of the mammalian immune system to *P. aeruginosa*

Innate immune defence molecules ^73, 74^, CD95-mediated apoptosis of epithelial cells ^75, 76^, and killing by polymorphonuclear neutrophilic granulocytes (PMNs) ^77^ play critical roles in fighting *P. aeruginosa*. PMNs are primarily recruited by chemokines to the diseased microenvironment. Upon chemokine binding, the chemokine receptor CXCR2 mediates the migration of the neutrophil to sites of inflammation and the chemokine receptor CXCR1 stimulates the production of reactive oxygen species to kill the *P. aeruginosa* bacteria ^78^. The clearance of the bacteria, however, should not be accompanied by excessive and systemic inflammation. Recent studies on the cytokines of the interleukin-17 (IL-17) family highlighted this delicate balance between antibacterial host response and inflammation-triggered organ pathology ^79– 81^. Through the use of a chronic murine pulmonary infection model, IL-17 cytokine signalling was found to be essential for mouse survival and the prevention of chronic infection with *P. aeruginosa* ^79^. On the other hand, IL-17A deficiency protected mice from an acute lethal *P. aeruginosa* lung infection ^80^. In the lung, IL-17A is released from numerous T-cell subtypes, innate lymphoid cells, and macrophages. IL-17A recruits inflammatory cells, but it also activates the airway epithelium to produce IL-17C. IL-17C increases the release of neutrophilic cytokines from alveolar epithelium and thus amplifies inflammation ^81^. IL-17C production is also directly induced by *P. aeruginosa.* Thus, epithelial cells activated by both the pathogen and the professional immune cells contribute to local and systemic inflammation during *P. aeruginosa* infection ^81^.

The IL-17 story demonstrates the context-dependent, subtle balance of harm and benefit of host defence mechanisms against *P. aeruginosa*. The ongoing race between pathogen and host has also resulted in highly specific host responses to individual bacterial components and vice versa. Some key mechanisms have recently been elucidated.

The secondary metabolite phenazine, for example, is recognised by the aryl hydrocarbon receptor AhR that triggers the recruitment of PMNs to the site of the bacterial insult ^82^, whereas the secondary metabolite pyocyanin induces the release of reactive oxygen species from mitochondria, which promotes the death of PMNs ^83^. These key cells of host defence not only eliminate *P. aeruginosa* by phagocytic killing but also trap and kill the microbes by neutrophilic extracellular traps (NETs), which are made up of DNA as the scaffold and neutrophilic granule components such as neutrophil elastase and myeloperoxidase ^84, 85^. The flagellum has been shown to be the main bacterial organelle that induces NET formation and thus triggers inflammation ^86^. Early growth phase *P. aeruginosa* are the strongest NET inducers. On the other hand, flagellar motility is downregulated and the flagellum is even lost during the course of chronic lung infections so that *P. aeruginosa* evades NET-mediated killing ^86– 88^.

If *P. aeruginosa* conquers a mammalian niche, it delivers the T3SS effector molecule ExoS directly into the cytosol of the host cell. In a mouse pneumonia model, phagocytes were targeted for injection of ExoS early during infection followed by injection of epithelial cells at later time points so that finally the pulmonary–vascular barrier was disrupted ^89^. This mechanism of stepwise inactivation of host defence cells first and the epithelial barrier thereafter makes it plausible that *P. aeruginosa* bacteria in the lung can gain access to the bloodstream and cause sepsis.

## Looking back forward

*P. aeruginosa* has been and still is one of the medically most relevant opportunistic pathogens in man ^2, 90, 91^. The management of eye ^92^ and burn wound ^93, 94^ infections has made considerable progress, but the acute ^95^ and chronic ^4, 5, 95– 99^ pulmonary infections continue to be associated with substantial morbidity and mortality.

Thanks to the application of omics technologies, we have gained insight into *P. aeruginosa*’s genome organisation and diversity ^8, 10, 11, 22, 100^, habitat-specific transcriptome ^101– 105^, proteome ^106– 108^, and metabolome ^108– 112^ and the co-evolution of *P. aeruginosa* with competitors in human habitats such as *Staphylococcus aureus* ^113– 115^. The function of hundreds of previously uncharacterised “conserved hypotheticals” ^11^ and the structure of secretory nanomachines have been resolved ^37, 41, 116– 121^ and knowledge has been gained about the complex regulation of the release of exopolysaccharides, secondary metabolites, and virulence effectors ^1^. In other words, during the last few years, we have learnt a lot about the modules of bacterial pathogenicity ^1, 122^. In contrast, progress has been rather slow in the field of antipseudomonal host defence. The mechanisms that mediate bacterial clearance without causing excessive immune pathology deserve further investigation if we want to improve the management of Pseudomonas pneumonia and sepsis ^123– 126^. *P. aeruginosa* is an extremely versatile microorganism, and it will continue to surprise us with as-yet-unappreciated modes of niche adaptation, lifestyle, and pathogenicity.
